# Phylogeography and postglacial expansion of the endangered semi-aquatic mammal *Galemys pyrenaicus*

**DOI:** 10.1186/1471-2148-13-115

**Published:** 2013-06-06

**Authors:** Javier Igea, Pere Aymerich, Angel Fernández-González, Jorge González-Esteban, Asunción Gómez, Rocío Alonso, Joaquim Gosálbez, Jose Castresana

**Affiliations:** 1 Institut de Biologia Evolutiva (CSIC-UPF), Passeig Marítim de la Barceloneta 37, Barcelona 08003, Spain; 2 Imperial College London, Silwood Park Campus, Ascot, Berkshire SL5 7PY, UK; 3 Departament de Biologia Animal, Universitat de Barcelona, Avinguda Diagonal; 4 Biosfera Consultoría Medioambiental S.L, Calle Candamo 5, Oviedo 33012, Spain; 5 Desma Estudios Ambientales S.L, Ukulu 11, Sunbilla, Navarra 31791, Spain; 6 Tragsatec, Área de Biodiversidad, Calle Julián Camarillo 6, Madrid 28037, Spain

**Keywords:** Conservation genetics, Introns, Mammals, Mitochondrial genes, Nuclear genes, Pyrenean desman, Niche modeling, Iberian Peninsula, Endemism

## Abstract

**Background:**

Species with strict ecological requirements may provide new insights into the forces that shaped the geographic variation of genetic diversity. The Pyrenean desman, *Galemys pyrenaicus*, is a small semi-aquatic mammal that inhabits clean streams of the northern half of the Iberian Peninsula and is endangered in most of its geographic range, but its genetic structure is currently unknown. While the stringent ecological demands derived from its aquatic habitat might have caused a partition of the genetic diversity among river basins, Pleistocene glaciations would have generated a genetic pattern related to glacial refugia.

**Results:**

To study the relative importance of historical and ecological factors in the genetic structure of *G. pyrenaicus*, we used mitochondrial and intronic sequences of specimens covering most of the species range. We show, first, that the Pyrenean desman has very low levels of genetic diversity compared to other mammals. In addition, phylogenetic and dating analyses of the mitochondrial sequences reveal a strong phylogeographic structure of a Middle Pleistocene origin, suggesting that the main lineages arose during periods of glacial isolation. Furthermore, both the spatial distribution of nuclear and mitochondrial diversity and the results of species distribution modeling suggest the existence of a major glacial refugium in the northwestern part of the Iberian Peninsula. Finally, the main mitochondrial lineages show a striking parapatric distribution without any apparent exchange of mitochondrial haplotypes between the lineages that came into secondary contact (although with certain permeability to nuclear genes), indicating incomplete mixing after the post-glacial recolonization. On the other hand, when we analyzed the partition of the genetic diversity among river basins, the Pyrenean desman showed a lower than expected genetic differentiation among main rivers.

**Conclusions:**

The analysis of mitochondrial and intronic markers in *G. pyrenaicus* showed the predominant effects of Pleistocene glaciations on the genetic structure of this species, while the distribution of the genetic diversity was not greatly influenced by the main river systems. These results and, particularly, the discovery of a marked phylogeographic structure, may have important implications for the conservation of the Pyrenean desman.

## Background

The genetic diversity patterns of species are a consequence of their evolutionary history (e.g. the existence of past refugia or vicariant geological events) and of contemporary constraints to dispersal (e.g. habitat fragmentation). These processes are expected to give rise to specific phylogeographic patterns [1-3], the detection of which can be useful to infer the relative importance of different evolutionary and ecological forces. Pleistocene glaciations have been among the major drivers in shaping the genetic structure of species, particularly in the Northern Hemisphere [4-7]. The isolation of populations in separate glacial refugia generated, first, a subdivision of the genetic pool of species into clearly distinct lineages. Moreover, subsequent colonization of new areas caused a particular pattern of genetic diversity in which past refugia retained maximum levels of genetic diversity whereas recently colonized regions became more homogeneous [4]. However, current barriers to gene flow may be more determinant in the genetic structure of species inhabiting naturally fragmented habitats [8] or in species that have very specific ecological requirements, such as aquatic organisms [1].

The Pyrenean desman (*Galemys pyrenaicus*) is a small semi-aquatic mammal endemic to the northern half of the Iberian Peninsula. It occupies streams of clean and cold flowing waters with shallow but permanent water levels throughout the year, an habitat generally found in mountain areas. Its distribution is highly dependent on the presence of larvae of benthonic macroinvertebrates that the desman captures underwater. Adaptations to the aquatic life include a highly-mobile protracted snout, large hindfeet and a long tail with stiff hairs [9,10]. Like many other specialists, the Pyrenean desman is an endangered species. For causes not clearly understood, it is undergoing significant declines across its whole geographic range. The situation has worsened during the last few years, particularly in the most southern populations, which have more Mediterranean climate. The decline of some populations has created a very fragmented distribution in this species [11]. The Pyrenean desman is legally protected in the four countries where it is present (Spain, Portugal, France and Andorra) and currently appears as “Vulnerable” in the IUCN Red List [12].

The Pyrenean desman forms part of the family Talpidae, which is included in the mammalian order Eulipotyphla (traditionally called Insectivora). Within Talpidae, the Pyrenean desman is placed within the subfamily Desmaninae together with the Russian desman (*Desmana moschata*), and therefore they are the only two extant representatives of this group of semi-aquatic mammals. Fossil data indicate that desmanines were much more diverse in the past [13,14] and the oldest fossil record is dated at 8.2 Myr [15]. The monophyly of desmanines is strongly supported by molecular data [16] and the divergence between both extant species has been estimated at around 10 million years (Myr) ago [17]. Therefore these two desman species are the last representatives of a unique lineage of specialist mammals that have experienced elevated extinction rates in the last few million years.

The Pyrenean desman is therefore an endemic, highly specialized, and relict species of great evolutionary and ecological interest. However, the genetic structure of the Pyrenean desman is yet to be investigated. Being a species with stringent ecological requirements, in which not all apparently favorable rivers are occupied [18], it is possible to hypothesize that the distribution of suitable habitats, very fragmented by their own nature, played a major role in structuring the genetic diversity of the species. For example, the genetic diversity could be partitioned, as in other organisms with strong aquatic requirements, according to major rivers or basins [1]. On the other hand, *G. pyrenaicus* is a polymorphic species in which two subspecies, *pyrenaicus* and *rufulus*, have been described according to differences in coloration and size. The validity of these subspecies and their distribution are still a matter of debate [19-21] but it has been postulated that these differences arose from geographic isolation during the Pleistocene glaciations [21]. Thus, the Pyrenean desman is a species of great interest on its own but it is also an ideal model to study how different ecological and evolutionary forces may have operated to establish the current distribution and genetic structure of species with strong ecological requirements.

To carry out a thorough genetic study of the Pyrenean desman, we first set up a noninvasive method of DNA extraction using droppings deposited on exposed rocks of the rivers it inhabits. Feces have previously been used to detect the presence of this elusive species [18,22] and represent a very valuable source of samples for genetic analyses across its whole distribution range. We favored a homogeneous sampling strategy in which samples were collected from as many localities as possible, rather than from discrete populations, to reduce biases in the delimitation of clusters and to better discern genetic diversity gradients [23]. Apart from feces, we also used tissue samples obtained from different biological collections as well as museum specimens. To assess the genetic diversity and the degree of connectivity between populations we used mitochondrial markers and nuclear introns. We show here how intron markers previously developed to be variable between closely related species and populations [24] can provide crucial information to study the evolutionary history of species. Our results allowed us to obtain, for the first time, important insights about the population history of the Pyrenean desman, and may have critical implications for the conservation of this endangered species.

## Methods

### Samples

Three types of samples of *G. pyrenaicus* were used for this study: feces, tissues obtained from different biological collections and museum samples (Additional file 1: Table S1). Fresh fecal samples were collected from different river localities, georeferenced and conserved in tubes containing absolute ethanol. To avoid using more than one fecal sample from the same individual, we only used sequences obtained from samples collected at least one kilometer apart, which is two to three times the typical home range of the Pyrenean desman [25,26]. Samples collected within that distance, but with different genotypes, were also used. This way, 69 fecal samples were included in the study. Moreover, tissue samples from 63 specimens were obtained from well-preserved specimens of different biological collections. Finally, the dataset was supplemented with 2 historical bone samples (a claw and a rib fragment) donated from the museum collection of the Doñana Biological Station.

### DNA extraction

Fecal samples were extracted using the QIAamp DNA Stool Kit (QIAGEN), following the manufacturer’s instructions, in a final elution volume of 50 μl of water. These extractions were carried out in a separated UV-irradiated area with dedicated equipment. Tissue samples were processed with QIAGEN DNeasy Blood and Tissue Kit, according to the manufacturer’s instructions, and eluted in 75 μl of water. When necessary, to ensure maximum tissue lysis, samples were incubated in a lysis buffer with proteinase K at 56°C overnight.

The extraction of the two museum bone samples was carried out in a dedicated ancient DNA laboratory. The samples were powdered and decalcified overnight in a 10 M EDTA solution at 37°C, followed by an overnight incubation in a lysis buffer with proteinase K and SDS at 56°C. The DNA was then extracted using a standard phenol-chloroform protocol [27] and finally concentrated using centricon columns.

### PCR of mitochondrial sequences in feces and tissue samples

All PCR reactions were set up in a dedicated PCR clean-room that is physically separated from post-PCR working areas and regularly decontaminated by UV-irradiation. For each sample, we amplified the complete cytochrome *b* gene (1140 bp) and a fragment containing 342 bp of the 5′ distal part of the D-loop, using *G. pyrenaicus* specifically-designed primers (see Additional file 1: Table S2). In addition, the cytochrome *b* gene of the Russian desman, *Desmana moschata*, was also amplified from a tissue sample of this species. For fecal samples, due to DNA degradation, the cytochrome *b* gene was usually sequenced in three overlapping fragments of 483, 278 and 516 bp, respectively. For fresh tissue DNA, the complete cytochrome *b* was amplified in a single PCR reaction.

PCR reactions were performed in a final volume of 25 μl, containing 2–4 μl of genomic DNA, 1 μM of each primer, 0.75 units of Promega GoTaq DNA polymerase and 17.5 μg of bovine serum albumin, under the following conditions: an initial denaturation of 2 min at 95°C, followed by 35 cycles of denaturation (30 s at 95°C), annealing (30 s at 54°C) and extension (30 s at 72°C). A 5-minute extension at 72°C was finally added. PCR products were revealed by electrophoresis in a 1% agarose SYBR-Safe (Invitrogen) stained gel.

### PCR of mitochondrial sequences of museum samples

Museum samples may contain degraded DNA due to chemical damage during the preservation process, thus incorporating induced mutations into some DNA molecules [28,29]. To prevent these artificial mutations to be eventually included in the recovered sequence, we obtained two independent estimates of the sequences of the museum samples.

Preliminary tests revealed that the sample IBE-C3161 (a rib fragment) had DNA with a similar concentration and quality than fecal samples. Thus, the protocols used for this sample were the same ones used for feces, except that two independent PCR reactions per fragment were performed so that we obtained two independent sequences.

The sample IBE-C3159 (a claw), on the other hand, was obtained from an individual captured in 1973 and had a much more degraded DNA. Therefore, the amplification of the cytochrome *b* and the D-loop was achieved using a two-step multiplex approach [30]. Two overlapping and independent sets of primers that covered the whole sequence of each mitochondrial marker were designed, the corresponding fragments ranging between 70 and 115 base pairs. For cytochrome *b*, two independents sets (A and B), each consisting of 8 non overlapping PCR products, were used, while the D-loop fragment was amplified using two smaller A and B sets of 3 and 2 PCR products, respectively. The 42 primer sequences are available upon request. In the first, multiplex step, all the primers of each independent set were used in a PCR reaction containing 5 μl of DNA, 2 units of Promega GoTaq DNA polymerase and 0.15 μM of each primer. The reaction conditions were as follows: initial denaturation at 94°C for 9 min and 30 cycles comprising denaturation at 94°C for 20 s, annealing at 54°C for 30 s and extension at 72°C for 30 s. Then, the second-step simplex PCRs were carried out for each individual fragment using a 1:20 dilution of the corresponding multiplex PCR product as the template, a concentration of 1.5 μM for each primer and following the same cycling conditions as for the first reaction. The 21 PCR reactions were directly sequenced and we obtained a single sequence. To obtain a second estimate of the sequence we repeated this multiplex PCR approach. Due to some difficulties with directly sequencing of some bands in the first PCR experiment, in the second round the fragments were cloned into the pstBlue-1 vector (Invitrogen). Three insert-containing plasmids were sequenced for each PCR fragment, thus obtaining a consensus sequence in which PCR errors revealed by the cloning process were disregarded.

Finally, for each sample, a comparison was made between the two sequences obtained via independent rounds of PCR reactions, and no differences were found. Thus, it seems that no extensive DNA damage had occurred during the preservation process of these two museum samples.

### PCR of nuclear sequences

A subset of 29 tissue samples that represented all mitochondrial lineages and covered the whole geographic distribution of the species was selected, and eight nuclear single-copy introns were sequenced from them. The amplified introns were *ACOX2-3*, *COPS7A-4*, *DHRS3-3*, *LANCL1-4*, *PRPF31-3*, *ROGDI-7* and *SMYD4-5*, chosen from the set described in Igea et at. [24], and an additional unpublished intron, *ACPT-4*, obtained during the filtering processes leading to this set [24]. The primers used are listed in Additional file 1: Table S3. PCR reactions were set up with the following conditions: an initial denaturation of 3 min at 95°C, and 32 cycles of denaturation (30 s at 95°C), annealing (30 s at variable temperatures; see Additional file 1: Table S3, for the temperature of each marker), and extension (60 s at 72°C). A 5-minute extension at 72°C was finally added.

Sequences of all the intronic markers were also amplified from *Desmana moschata* and a representative of Talpinae (*Talpa occidentalis*), following procedures similar to those described above.

### Sequencing

All PCR products were purified using ExoSAP-It (Affymetrix) and sequenced in both directions using the original PCR primers with BigDye v3.1 at different sequencing services. Sequences were inspected, trimmed and assembled using Geneious Pro (Biomatters Ltd.).

### Phylogenetic analyses

The cytochrome *b* and D-loop sequences of the 134 *G. pyrenaicus* samples were concatenated for further analyses. The optimal model of sequence evolution was determined using the Akaike Information Criterion with jModeltest version 0.1 [31]. The resulting model was the Hasegawa-Kishino-Yano (HKY) with among-site rate variation assuming a gamma distribution (Γ) and a proportion of invariable sites (I). Using this model, a maximum-likelihood phylogenetic tree was reconstructed with PhyML version 3.0 [32]. From this tree, a haplotype genealogy was generated using Haploviewer 1.0 [33]. The phylogenetic relationships among the *G. pyrenaicus* mitochondrial sequences were also inferred using a Bayesian approach, as implemented in BEAST 1.6.2 [34]. Previously, a molecular clock test was performed with PAUP* version 4.0b10 [35] by estimating the likelihood of the PhyML topology with and without forcing a molecular clock. A likelihood-ratio test [36] indicated that the molecular clock hypothesis could not be rejected. Therefore, a strict molecular clock was used in BEAST and, as above, a HKY + Γ + I evolution model was set. For the tree prior, a coalescent constant population size model was used. All sites were used in a single partition but similar results were found when we set one partition per codon position and another one for the D-loop (not shown; results were similar with and without partitions likely due to the low genetic divergences within the species). The Markov chain was run for 50 million generations and sampled every 1000 generations. Convergence was checked with the BEAST utility Tracer, ensuring that all effective sample size values were greater than 200. In addition, we ensured that similar results were obtained across multiple runs. We removed the first 10% of the samples as burn-in and obtained the subsequent maximum clade credibility summary tree with median node heights using the BEAST utility TreeAnnotator.

For the heterozygous nuclear sequences, distinct haplotypes were manually obtained since the sequences contained only one heterozygous position. Haplotype genealogies were then generated for each marker using Haploviewer from the corresponding PhyML tree.

### Genetic diversity, demographic and genetic differentiation analyses

Nucleotide and haplotype diversity parameters were estimated using DnaSP version 5 [37]. Signals of departure from neutrality, which could be interpreted as past population expansions, were tested using Tajima’s D [38], Fu’s F_s_[39] and R_2_[40] statistics. Genetic differentiation among groups (one level) was assessed by analysis of molecular variance (AMOVA) of the mitochondrial sequences using pairwise differences, with Arlequin 3.5 [41].

Correlation of genetic and geographical distances was assessed with a Mantel test using the program Alleles In Space 1.0 [42]. Genetic barriers across the *G. pyrenaicus* distribution area were determined with the Monmonier’s Maximum Difference algorithm [43], which identifies the greatest genetic distance between any two locations, also using Alleles In Space [42]. For this analysis, we used raw genetic distances calculated from the concatenated mitochondrial data and the corresponding geographical coordinates, setting for only one barrier to be detected.

Mitochondrial genetic diversity was estimated at each sampling location by using all sequences collected within 1 degree (approximately 100 km) of the location. This area allowed the estimation of genetic diversity from a good number of samples at each point, yet the resolution was good enough to distinguish regional differences in genetic diversity. In addition, centering the measurements around each sampling location, rather than using a fixed grid, allowed the efficient grouping in less sampled areas. To avoid inflating genetic diversity due to lineages in secondary contact, only sequences belonging to the same lineage were used for each locality but, for comparison, additional analyses were performed with mixed lineages. For each subset of sequences around a location with more than two samples, nucleotide diversity (π) was estimated. A regularly spaced grid of π values was then interpolated and a contour map was constructed using Surfer 10.2 (Golden Software Inc.).

### Estimation of the time to the most recent common ancestor (MRCA) of the mitochondrial sequences

Since no reliable multiple fossil calibrations close to *G. pyrenaicus* could be used to date the mitochondrial lineage splits, we had to rely on more external mammalian fossil data. However, in trees of divergent mammalian groups, mitochondrial genes are saturated whereas nuclear genes are more adequate [44]. Therefore, the estimation of the time to the MRCA of the *G. pyrenaicus* mitochondrial haplotypes was done in two steps. First, we obtained an accurate calibration of the *G. pyrenaicus– D. moschata* split from a Bayesian nuclear tree of Laurasiatherian mammals with multiple fossil data. For this analysis, we used the eight introns sequenced in this study from *G. pyrenaicus*, *D. moschata* and *T. occidentalis,* as well as the corresponding orthologous sequences of the following Laurasiatherian mammals with genomes available in the Ensembl database [45]: *Felis catus*, *Canis familiaris*, *Pteropus vampyrus*, *Equus caballus*, *Bos taurus*, *Tursiops truncatus* and *Sus scrofa*. For some of the species, not all the orthologous introns were available, resulting in 7.5% of missing data. Intron alignments were built with MAFFT using the L-INS-i accuracy-oriented method [46]. Gblocks was subsequently applied with relaxed parameters to discard poorly aligned regions [47]. These eight intron alignments were included as independent partitions in a BEAST analysis. The appropriate substitution model (as suggested by jModeltest) and an uncorrelated lognormal (UCLN) relaxed molecular clock were chosen for each partition. A relaxed clock was used since a likelihood-ratio test performed as above rejected the strict molecular clock for all introns. A Yule speciation model was used as tree prior. As calibrations, we used multiple mammalian fossil constraints previously compiled for key nodes [48], which include “hard” minimum and “soft” maximum constraints, thus making time estimations less sensitive to the parameters of the prior distributions [49]. Using these data, we set lognormal prior distributions as follows: the offset was defined by the hard minimum, the mean in real space was adjusted so that the upper 95th percentile of the probability density distribution was coincident with the soft maximum, and the standard deviation was set to 1. The analysis was run for 100 million generations, and 10% of the trees were discarded as burn-in before computing the corresponding maximum clade credibility tree using median node heights. In addition, following Drummond *et al.*[50], we evaluated the interaction among different calibration priors by running BEAST analyses without sequence data for the same number of generations. It was verified that the distributions of effective priors were included within the distributions of the corresponding priors, discarding the existence of unexpected interactions between priors.

In a second step, the resulting posterior distribution of the age of the *G. pyrenaicus – D. moschata* split was used in a subsequent analysis using only talpid cytochrome *b* sequences to estimate the time to the MRCA of the *G. pyrenaicus* mitochondrial sequences. In addition to all sequenced cytochrome *b* haplotypes of *G. pyrenaicus* (35 unique haplotypes) and *D. moschata*, we obtained from GenBank complete cytochrome *b* sequences of representative Talpinae species (75 haplotypes belonging to 18 species). Talpinae is a sister group to the *G. pyrenaicus – D. moschata* group (Desmaninae) and therefore it is the most adequate outgroup. In the BEAST analysis of these 111 sequences, the TrN + Γ + I model of sequence evolution was chosen following jModeltest, and a UCLN clock was assumed (since a likelihood-ratio test rejected the strict molecular clock for these talpid sequences). Due to the divergence of cytochrome *b* within the talpids family, particularly in third-codon positions, the sites were partitioned according to the three codon positions, so that each partition had its own model parameters. For the tree prior, a coalescent constant population size model was used since the node of main interest was intra-specific. The desmanines split was calibrated using a normal distribution with the mean and standard deviation taken from the results of the previous analysis of Laurasiatherians. Running conditions were the same as above.

### Species-distribution modeling

In order to develop a distribution model of *G. pyrenaicus*, occurrence data were taken from the species distribution atlases of Spain [11], Portugal [22] and France [51]. Coordinates of the records were obtained from the respective atlases or from the Global Biodiversity Information Facility (http://www.gbif.org/). For each record, the center of the corresponding 10×10 km UTM square was taken, resulting in a total of 680 unique data points. The study area was defined between 39 and 44° latitude, and −10 and 4° longitude, encompassing the whole distribution area of *G. pyrenaicus* plus additional areas of potential dispersal, suitable for the selection of background data [52].

The 19 BioClim climatic variables [53], which represent summaries of means and variations in temperature and precipitation, plus altitude, were downloaded from the WorldClim global climate database version 1.4 at a spatial resolution of 2.5 arc-minutes (http://www.worldclim.org). Climatic variables were downloaded for present conditions and for the Last Glacial Maximum (LGM). For the latter, both the Community Climate System Model (CCSM) and the Model for Interdisciplinary Research on Climate (MIROC) were used. Colinearity among the climatic variables for present conditions was analyzed by means of pairwise correlations using 1000 randomly selected points from the area of interest. After removing variables with correlation coefficients greater than 0.9, we retained the following 11 variables: BIO1 (Annual Mean Temperature), BIO2 (Mean Diurnal Range), BIO3 (Isothermality), BIO4 (Temperature Seasonality), BIO5 (Max Temperature of Warmest Month), BIO6 (Min Temperature of Coldest Month), BIO8 (Mean Temperature of Wettest Quarter), BIO9 (Mean Temperature of Driest Quarter), BIO12 (Annual Precipitation), BIO14 (Precipitation of Driest Month), and BIO15 (Precipitation Seasonality).

To predict the potential distribution of the species in current conditions and in the LGM we used Maxent version 3.3.3 [54], which outputs a model with relative occurrence probability of a species within the grid cells of the study area. We used default settings, except that the model was run with 100 crossvalidate replicates, taking the mean values of the probabilities of presence. Accuracy of the model was tested using 75% of the presence data to train the model and 25% to test the model. The area under the receiver operating characteristic curve (AUC) for the test data resulted in a value of 0.824, which is considered to correspond to a useful predictive model [55]. Finally, this distribution model was used to predict the potential distribution of the species during the LGM using the CCSM and MIROC models. However, the MIROC model predicted very mild climatic conditions for the LGM in this part of the world and thus the distribution predicted for the Pyrenean desman in the LGM was very similar to the present distribution. The presence of many cold-adapted species in the Iberian Peninsula during the LGM [56] is not congruent with this model and therefore it was not used alone. When we analyzed both CCSM and MIROC models to estimate the minimum common area under both models [57], the resulting potential distribution was very similar to the results of the CCSM model, since this is the most restrictive model (not shown). Thus, only the CCSM model was used for the final analyses.

## Results

### Mitochondrial phylogeographic analysis

**Figure 1:**
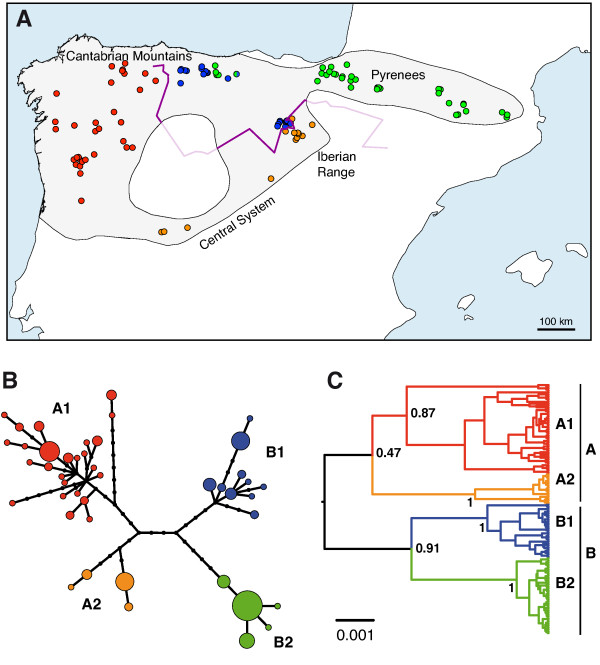
**Phylogeographic analysis of the mitochondrial sequences of *Galemys pyrenaicus*.** (**A**) Map of the northern part of the Iberian Peninsula showing the 134 samples of *G. pyrenaicus* used in this study. The grayed area represents the historical species distribution according to different sources. Names of mountain ranges mentioned in the text are shown. Each sample is represented by a circle, but a few samples with the same coordinates cannot be discerned. Colors of the samples indicate the four different mitochondrial lineages recovered in the phylogenetic analyses (A1, A2, B1 and B2). The single locality with two samples belonging to two different lineages (B1 and B2) is shown with both corresponding colors. The purple line indicates the genetic barrier identified by the Monmonier’s Maximum Difference algorithm (the intersection with the species distribution area is shown with stronger color). (**B**) Haplotype genealogy of the concatenated mitochondrial sequences based on a maximum-likelihood tree. Circles represent haplotypes, with size being proportional to the number of individuals, and black dots representing intermediate, unsampled haplotypes. (**C**) Bayesian tree of the same sequences. Posterior probabilities for relevant clades are shown. The scale bar represents 0.001 substitutions/position.

We used 134 samples of *G. pyrenaicus* from 115 different localities covering a large part of the species distribution range and all important river basins (Figure 1A). For each sample, the complete cytochrome *b* gene and a D-loop fragment were sequenced and concatenated to make a total of 1482 bp per individual. The haplotype genealogy reconstructed from a maximum-likelihood tree (Figure 1B) and a Bayesian molecular-clock tree (Figure 1C) of the sequences revealed two large groups, A and B, each subdivided into two further groups to give a total of four distinct lineages: A1, A2, B1 and B2. In addition, these lineages presented a prominent parapatric distribution (Figure 1A). Some mixing can only be observed in the contact zone between lineages B1 and B2 (eastern part of the Cantabrian Mountains). However, the most remarkable pattern occurs in the contact zone between lineages A2 and B1 (in the Iberian Mountain Range; Figure 1A), where individuals corresponding to both lineages are separated by a narrow band of a few kilometers without any exchange of haplogroups, at least in the individuals sampled so far.

**Figure 2:**
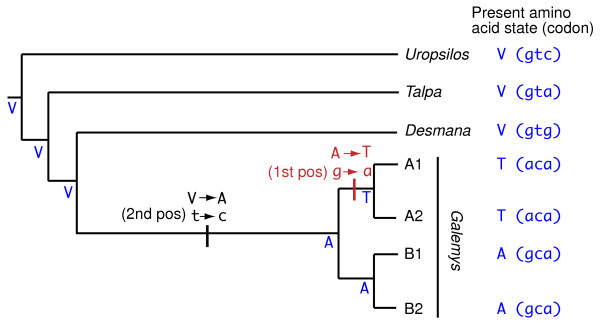
**Parsimonious reconstruction of the amino acid changes along the phylogeny of cytochrome *b *in talpids.** Present state of the amino acids and codons in position 329 of the cytochrome *b* protein is shown in blue color for several talpid species, including *Desmana* and *Galemys*. Deduced ancestral amino acid states are shown for each node in blue color. The two non-synonymous changes deduced in this position are represented by a vertical bar in the corresponding branches. For every change, the codon and amino acid changes are shown. The change shared by *G. pyrenaicus* lineages A1 and A2 is shown in red color.

The position of the root of the phylogeny of the mitochondrial lineages is important to interpret the evolution of the desman populations. Unfortunately, the sequence of the closest species, *D. moschata*, is too distant to be used as an adequate outgroup in an unrooted analysis such as the maximum-likelihood genealogy of Figure 1B. On the other hand, the rooted tree obtained from the Bayesian analysis of the *G. pyrenaicus* sequences (Figure 1C) renders a good posterior probability for the grouping of lineages B1 and B2 (0.91), but very low for the grouping of A1 and A2 (0.47). However, an examination of the protein sequences deduced from the cytochrome *b* gene (including the *Desmana* sequence, which is more informative as an outgroup at the protein level) revealed that one of the few amino acid changes that occurred along this phylogeny is shared by all individuals of lineages A1 and A2 (Figure 2). Since a non-synonymous substitution is a very rare change, it likely occurred only once and we thus place it in the (short) lineage leading to the common ancestor of A1 and A2, after the separation of the common ancestor of all lineages. Therefore, the protein sequences support the topology of Figure 1C as the most likely one for the relationship among the four mitochondrial lineages.

In agreement with this root, which indicates that the deepest divergence occurred between groups A and B, the Monmonier’s Maximum Difference algorithm [43] identified the greatest genetic distance in the two contact zones between these two composite groups (Figure 1A).

The divergence between the lineages is quite shallow, with 1% mean differences (p distance) between groups A and B and 0.8% mean differences in the comparisons of both A1 with A2 and B1 with B2.

### Mitochondrial genetic diversity

The overall genetic diversity of the Pyrenean desman measured with the concatenated mitochondrial sequences is relatively low (Table 1), with a value of nucleotide diversity (π) of 0.0073 (for comparison purposes, π is 0.0060 for the cytochrome *b* gene alone). When this value was calculated for each lineage, important differences were found among them, with one order of magnitude difference between the lineages with the highest (A1: 0.0036) and lowest (B2: 0.0004) nucleotide diversity values. In addition, a significant signal of population expansion was found only for the lineage A1 with the R_2_ and Fu’s F_s_ statistics but not with Tajima’s D. As previously shown [40], while Tajima’s D may not be able to detect population expansions under certain conditions, both R_2_ and Fu’s F_s_ have been shown to detect deviations from a constant population size in a wide variety of situations.

**Table 1:** **Mitochondrial genetic diversity and population expansion statistics of the concatenated complete cytochrome*b*sequence and a D-loop fragment of*Galemys pyrenaicus*calculated for the whole species and for the 4 mitochondrial lineages**

	**Whole species**	**A1**	**A2**	**B1**	**B2**
**N**	134	48	16	29	41
**S**	72	40	7	15	4
**h**	44	25	4	10	5
**Hd**	0.935	0.927	0.592	0.842	0.534
**π**	0.0073	0.0036	0.0016	0.0024	0.0004
**π (Tissues)**	0.0070	0.0038	0.0016	0.0028	0.0004
**π (Feces)**	0.0071	0.0029	0.0002	0.0024	0.0004
**Tajima’s D**	−0.545	−1.399	0.416	−0.202	−0.832
**R**_**2**_	0.074	0.062 (*)	0.163	0.112	0.083
**Fu’s F**_**s**_	−7.725	−10.458 (*)	1.89	−0.872	−1.645

N = number of sequences; S = number of segregating sites; h = number of haplotypes; Hd = haplotype diversity. Significant (p < 0.05) deviation from the neutral model is shown with an asterisk.

The use of feces to obtain genetic data could lead to an underestimation of genetic diversity values if several samples of the same individual are used. Our choice of using only feces separated at least 1 km should prevent this problem but, for completeness in the phylogeographic analyses, we also included a few samples within that distance when haplotypes were different (see Methods). To test if this approach of selecting feces generated an unbiased collection of samples, we calculated nucleotide diversity for the 69 fecal samples and the 65 tissues separately (Table 1). The results were very similar for both sample sets (0.0070 and 0.0071 for tissues and feces, respectively) and very similar to the whole set. When the four lineages were separately analyzed to test for differences between types of samples, nucleotide diversity values were also very similar in tissues and feces except for the lineage A2 (most likely due to the small sample size of this lineage). These results indicate that our sampling scheme for collecting feces did not distort the genetic diversity results and, therefore, added important information for the genetic study of the species.

**Figure 3:**
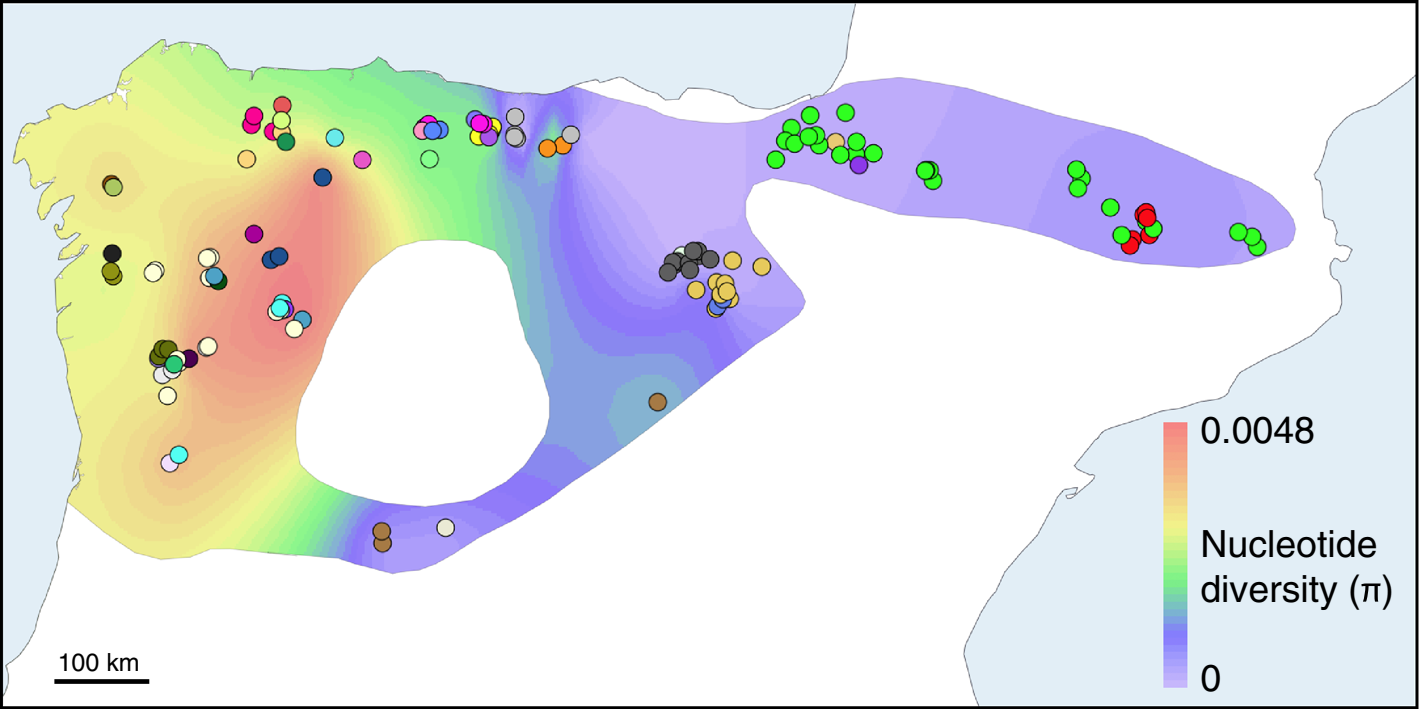
**Contour plot of genetic diversity (π) of *Galemys pyrenaicus.***Only samples of the same lineage were considered for calculating π at each locality. Colors of the contour plot indicate interpolated genetic diversity. The contour plot is only drawn for the species distribution area. Sample points have been randomly repositioned within a circle of 5 km radius to reveal samples of the same location. A different color is used for each haplotype and therefore a greater variety of colors in an area also indicates higher genetic diversity.

The contour map derived from the π values of the samples around each locality (Figure 3) clearly shows maximum levels of genetic diversity in the NW of the Iberian Peninsula. From this region, genetic diversity gradually decreases towards the eastern parts of the desman distribution. In this plot, only samples of the same lineage were considered for calculating π at each locality. When, for comparison, samples from lineages in secondary contact were also considered, the contour plot shows, as expected, the highest genetic diversity in the contact zones (Additional file 1: Figure S1).

**Figure 4:**
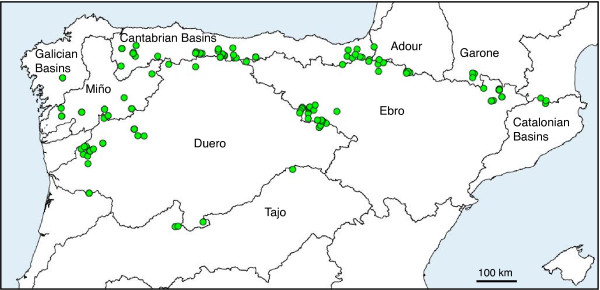
**Groups of *Galemys pyrenaicus *samples for the AMOVA analysis.** Samples were grouped by main drainage basins, as indicated by the drainage boundaries drawn in the map. Exact grouping of each sample is indicated in Additional file 1: Table S1.

We also analyzed the partition of genetic diversity by means of an AMOVA analysis. When we grouped the different desman samples by main drainage basin (Figure 4) we found a total of 32% of the genetic variation attributable to the grouping by major river systems. However, this value could be inflated by a strong correlation of genetic and geographical distances (Mantel test: r = 0.50; p = 0.001), which indicates that a pronounced pattern of isolation by distance is present in this system [58]. The A1 lineage occupies a smaller area and does not have a strong genetic structure, and therefore the isolation by distance effect is much smaller (Mantel test: r = 0.15; p = 0.017). If the AMOVA analysis is restricted to this lineage, only 15.6% of the genetic variation was explained by the grouping of populations by major river systems.

### Nuclear genetic diversity

**Figure 5:**
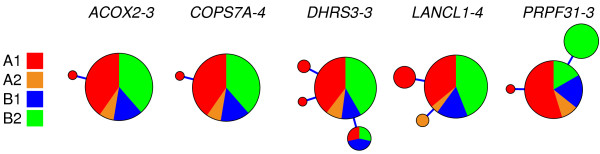
**Haplotype genealogies of the five introns that show some variability in *Galemys pyrenaicus*.** The size of the circles is proportional to the number of individuals. Colors indicate the four different mitochondrial lineages (A1, A2, B1 and B2) to which the specimens belong.

For 29 desmans, we sequenced eight nuclear introns, totaling 3256 bp per individual [24]. The suitability of these introns was indicated by the large number of differences found between the *Galemys* and *Desmana* sequences (Additional file 1: Table S3), discarding that they were subjected to functional conservation in the desmanines lineage. However, the analysis of these introns revealed that only five of them were variable within *G. pyrenaicus*, and with a very low number of alleles for each locus (Figure 5). As a consequence, the average genetic diversity of the eight introns was very low (π = 0.00034; Table 2). In addition, among the 232 sequenced introns, only 10 were heterozygous (average heterozygosity = 0.043), each with a single heterozygous position. To analyze the nuclear diversity across space we used the mitochondrial lineages as surrogates of populations due to the strong correspondence between geographic regions and mitochondrial lineages. Again, the highest average nucleotide diversity was found in the samples belonging to the A1 mitochondrial lineage (Table 2).

**Table 2:** **Nuclear genetic diversity (π) of the eight introns of*Galemys pyrenaicus*calculated for the whole species and for the four mitochondrial lineages**

**Intron**	**Whole**	**A1**	**A2**	**B1**	**B2**
***ACOX2-3***	0.00009	0.00022	0	0	0
***ACPT-4***	0	0	0	0	0
***COPS7A-4***	0.00005	0.00013	0	0	0
***DHRS3-3***	0.00122	0.00155	0	0.00206	0.00067
***LANCL1-4***	0.00049	0.00075	0.00127	0	0
***PRPF31-3***	0.00087	0.00017	0	0	0.00093
***ROGDI-7***	0	0	0	0	0
***SMYD4-5***	0	0	0	0	0
**Average**	0.00034	0.00035	0.00016	0.00026	0.00020

**Figure 6:**
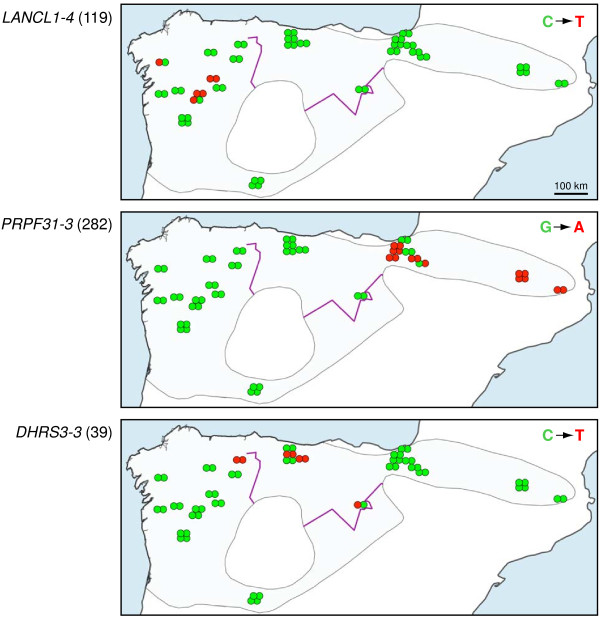
**Map showing the geographic distribution of the variants of 3 SNPs.** Nucleotide variants are shown for position 119 of intron *LANCL1-4*, position 282 of intron *PRPF31-3* and position 39 of intron *DHRS3-3*. For each specimen, the alleles of both chromosomes are represented with two adjacent points. The derived mutation (as deduced from the *Desmana* outgroup) is shown in red. Some points have been latitudinally shifted to reveal specimens of the same location. The purple line indicates the genetic barrier identified by the Monmonier’s Maximum Difference algorithm (only the intersection with the species distribution area is shown).

A comparison of these introns with the *Desmana* orthologs allowed us to establish the derived mutation in the *G. pyrenaicus* SNPs. Although most mutations were confined to one individual, three of them were sufficiently spread within the species range to be informative about connectivity among populations. While two of these variants were restricted to a single population (or mitochondrial lineage), one of them (position 39 of intron *DHRS3-3*) was present in at least three of these lineages: A1, B1 and B2 (Figure 6). Consequently, this variant crosses at least the Cantabrian Mountains contact zone.

### Estimation of the time to the MRCA of the mitochondrial sequences

**Figure 7:**
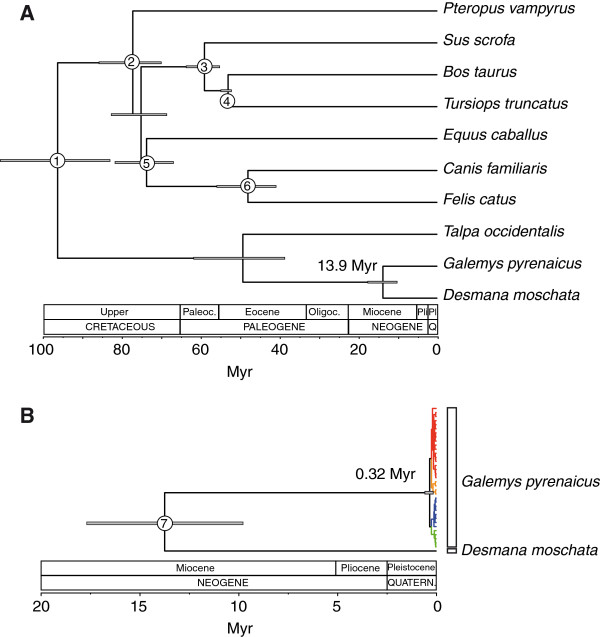
**Dating analysis of the *Galemys pyrenaicus *sequences.** (**A**) Bayesian dating analysis of mammalian sequences used to estimate the divergence time of *Galemys* and *Desmana*. Fossil constraints were used for the nodes corresponding to Laurasiatheria (1), Ferungulata (2), Cetartiodactyla (3), Cetruminantia (4), Zooamata (5) and Carnivora (6). Grey bars on each node represent the 95% HPD interval of the date. (**B**) Bayesian dating analysis of *G. pyrenaicus* mitochondrial sequences using the *Galemys*-*Desmana* split as calibration point. Colors indicate the four mitochondrial lineages of *G. pyrenaicus* recovered in the phylogeographic analysis. Outgroup sequences belonging to Talpinae are not shown.

The divergence time of *Galemys* and *Desmana* using eight intron sequences and multiple fossil constrains (Additional file 1: Table S5) was 13.9 Myr ago (Figure 7A). This date predates the oldest fossil record of Desmaninae at 8.2 Myr [15] and therefore extends the origin of this clade a few million years back. However, both dates are congruent since incompleteness of the fossil record could explain the lack of fossil desmanines older than 8.2 Myr. Our divergence time estimate and its standard deviation were introduced in a subsequent Bayesian analysis of cytochrome *b* sequences of *Galemys*, *Desmana* and other talpid species used as outgroups. The obtained cytochrome *b* evolutionary rate was 0.0224 substitutions/position/Myr and the resulting time to the MRCA of all the mitochondrial sequences, which represents the split time of the A and B groups, was 0.32 Myr, with a 95% highest posterior density (HPD) interval of 0.15 - 0.56 Myr, clearly within the Middle Pleistocene (Figure 7B). The estimation of the split time of the A1 and A2 lineages was 0.23 Myr (HPD= 0.10 - 0.40) and the divergence of the B1 and B2 lineages was 0.23 Myr (HPD = 0.10 - 0.42), also in the Middle Pleistocene. These dates represent the coalescence of the different mitochondrial lineages but the populations could have diverged at a much more recent time, that is, these dates establish the upper limit for the separation of the most divergent desman populations.

### Species distribution modeling in the LGM

**Figure 8:**
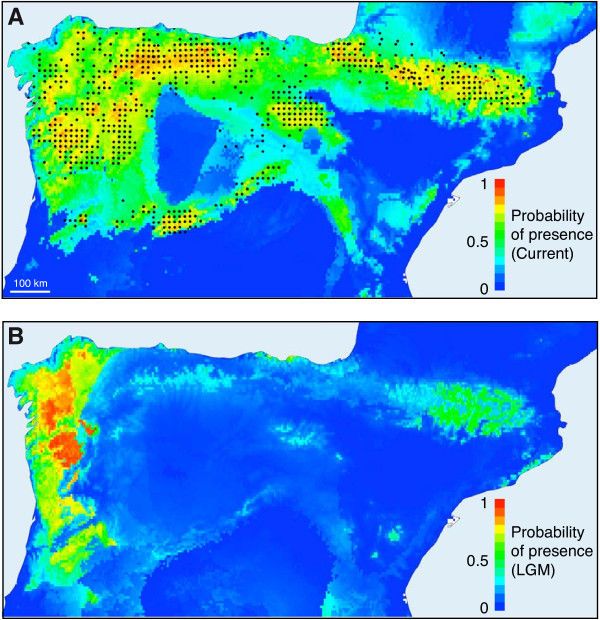
**Species-distribution modeling of *Galemys pyrenaicus*.** (**A**) Potential distribution of *G. pyrenaicus* as estimated by Maxent for present time. The blacks dots represent occurrence data used for this analysis. Colors indicate probability of presence. (**B**) Potential distribution of *G. pyrenaicus* during the Last Glacial Maximum.

To study the relationship between the conspicuous genetic diversity gradient found in the Pyrenean desman (Figure 3) and possible glacial refugia, we built a species distribution model based on the known-presence localities of *G. pyrenaicus* (Figure 8A). When this model was projected to the conditions of the LGM, we found that the maximum probabilities of potential presence occurred again in the NW part of the Iberian Peninsula (Figure 8B), in notable coincidence with the area of contemporary greater genetic diversity of the species (Figure 3). Although with lower probabilities, other isolated areas of potential presence in the LGM were also found (Figure 8B).

## Discussion

### Dating analysis of the mitochondrial lineages

We have been able to gather a number of solid pieces of evidence that show that the evolutionary history of *G. pyrenaicus* and the genetic structure of its populations were strongly influenced by the Pleistocene glaciations. Remarkably, the phylogeny of the mitochondrial genes exhibits a pronounced geographic pattern, with the presence of four lineages (grouped into two main phylogroups) that have a marked parapatric distribution. However, this structure is not by itself proof of the effects of glaciations as it may predate the Pleistocene. Therefore it is important to obtain an accurate dating of the splits of these lineages. Our dating approach allowed us to estimate that the split of the two most divergent mitochondrial phylogroups occurred 0.32 Myr ago and that the subsequent divergence of the two pairs of lineages concomitantly took place at around 0.23 Myr ago. Since sequence coalescence must be older than the population split, these dates represent the upper limit at which the desman populations started to diverge. Therefore, given these Middle Pleistocene lineage split times, it is very likely that the four desman populations evolved in four isolated glacial refugia, supporting the importance of the Pleistocene glaciations in the population structure of this species. Most probably, the populations started to diverge during earlier phases of the glacial periods and not necessarily in the last glaciation, explaining the deep mitochondrial divergences observed [59,60].

Since we did not have reliable fossils in desmanines, we had to use more external calibrations of mammals for our dating analysis. This analysis benefited from the nuclear introns, which allowed us to reconstruct a Bayesian tree of mammals calibrated with multiple fossils and to estimate the divergence date of *Galemys* and *Desmana*. The obtained date at 13.9 Myr was quite adequate to calibrate, in a subsequent step, the mitochondrial gene tree. On the one hand, this date is not as old as to present problems of saturation. On the other hand, it is not as recent as to suffer from the problems of coalescence, which can be exacerbated when dating very recent nodes of a gene tree (< 10 Myr) [61]. This calibration date was then introduced into a phylogenetic tree of the cytochrome *b* of talpids and, from this calibrated tree, we estimated the divergence time of the main *Galemys* mitochondrial lineages at 0.32 Myr. The evolutionary rate resulting with this approach for cytochrome *b* was 0.0224 substitutions/position/Myr. Although this evolutionary rate is line with those obtained for other mammalian groups [62,63], different dating approaches have led to much higher rates [64-66]. Also, actual quantification of mutation accumulation from pedigree data has shown more elevated evolutionary rates in mitochondrial genes, at least in humans [67]. It has been suggested that the possible existence of mutational hotspots and other problems [64] may cause that evolutionary rates can only be properly estimated in recent branches of a phylogeny. However, the extent of this effect is contentious [68,69]. Actually, it has been shown more recently that, in fact, lack of consideration of coalescence of ancestral polymorphisms in recent calibrations [61,70] or the use of too simple evolutionary models [71] may lead to altered results in dating analyses. Our approach included a calibration date in which coalescence should be negligible for usual population sizes in mammals [61] and we used a codon-partitioned model, which should avoid these problems. Nonetheless, an increase in the rate that we estimated for *Galemys* would only reduce, in the equivalent proportion, the split time of the mitochondrial lineages. Since our main purpose in this part of the work was to test if the separation of the mitochondrial lineages occurred in the Pleistocene, any increase in this rate would still support the Pleistocene split of the *G. pyrenaicus* mitochondrial lineages.

Although introns were very useful for obtaining the *Galemys*-*Desmana* split time in the first step of our dating analysis, the low variability of these sequences (Table 2) did not allow us to use them in a multilocus dating analysis for the second step, which would have permitted a direct estimation of the population splits in a species tree framework. In fact, the lack of accumulated differences at the nuclear level between the four desman populations may indicate more recent separations than the ones indicated by the mitochondrial genes. Further studies with additional nuclear data will help to resolve these issues.

### Pleistocene evolution of the Pyrenean desman populations

**Figure 9:**
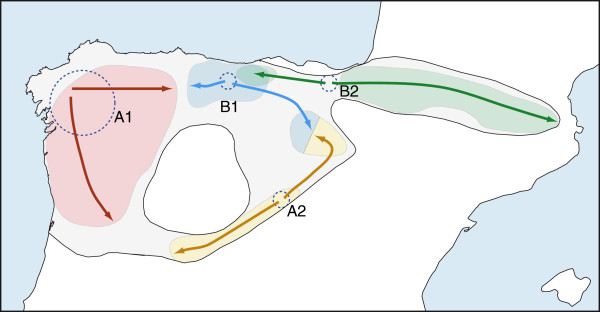
**Schematic representation of the evolutionary history of *Galemys pyrenaicus*.** The grayed area represents the historical species distribution. Sampled areas of the four mitochondrial lineages are shown in different colors. Hypothetical positions of glacial refugia are illustrated with dotted circles within the current distribution of each mitochondrial lineage. The size of the circles represents the relative importance of the refugia. Arrows indicate colonization routes from these refugia.

From the four inferred glacial refugia, other areas of the current distribution range would have been subsequently colonized during the Holocene, as depicted in a schematic scenario of the evolution of the desman populations (Figure 9). Given the strong geographic pattern of the four mitochondrial lineages of *G. pyrenaicus*, it is possible to speculate about the relative locations of the refugia where they evolved. Different independent pieces of data support that the NW of the Iberian Peninsula served as the major glacial refugium for the desman populations. In particular, we found the greatest genetic diversity of the species in the northwestern part of the Iberian Peninsula and much more homogeneous populations towards the eastern parts of the desman distribution. In addition, distribution models of the Pyrenean desman projected to the conditions of the LGM predicted the highest probabilities of potential presence in the same northwestern area. The coincidence between the highest genetic diversity and the predictions of potential presence in the LGM in the same area is very remarkable, but it is the expected result from a classical phylogeographic scenario in which only part of the genetic pool from the glacial refugia colonized new areas. Furthermore, a signal of population expansion was detected in the northwestern mitochondrial lineage. This area was a likely glacial refugium for other species that also depend on aquatic habitats such as the golden-striped salamander [72], so this region probably preserved optimal temperature and pluviometric conditions for species with these particular requirements during the successive glacial cycles.

An additional refugium situated in the Iberian Range or the Central System could have given rise to the populations of lineage A2, but the location of this refugium remains very speculative due to the scarcity of data for this lineage. Given the genetic proximity of this lineage to the northwestern A1 lineage, the refugium could have been situated somewhere in the Central System rather than in the more distant Iberian Range. If this were the case, part of the Iberian Range (Cameros, Urbión and Cebollera Mountains) would have been recently colonized. However, the dispersal, at least of females, would not have progressed towards the northwestern parts of the Iberian Range (Demanda Mountains).

Lineage B1 could have evolved in a refugium in the Cantabrian Mountains, where the maximum genetic diversity for this lineage was found. From this refugium, the species would have colonized the northwestern part of the Iberian Range (Demanda Mountains). However, at least the females would not have continued the dispersal towards more southeastern parts of the Iberian Range (Cameros, Urbión and Cebollera Mountains). Dispersal of lineage B1 from its putative refugium towards the western parts of the Cantabrian Mountains would have also been limited, and, at least the females, would not have crossed to the areas occupied by lineage A1.

Despite potentially suitable refugia within the Pyrenees [73], the Pyrenean populations of desmans, which belong to lineage B2 and are highly homogeneous genetically, must have originated from a distant refugium after a severe bottleneck. This refugium could have been placed towards the middle of the distribution this lineage, such as in the Basque Mountains, as previously suggested according to the current distribution of the species [74]. The colonization of the Pyrenees must have been quite recent and likely occurred very quickly and through an important bottleneck, as deduced from the very low nuclear and mitochondrial diversity observed in the desmans of these mountains. From this putative refugium, the populations of this lineage also dispersed in other directions, towards the areas currently occupied by lineage B1, so that there is currently mixing of both B lineages in some rivers. The areas proposed here as likely glacial refugia for lineages B1 and B2, in the Cantabrian Mountains and the Basque Mountains, respectively, have been previously postulated to maintain potential refugia for many other species associated to humid climates, including mammals [75,76] and plants [77].

The distribution of mitochondrial lineages and their variability allowed us to infer a clear postglacial expansion of the desman populations from the refugial areas. This range expansion should be in principle accompanied by an increase in population size, but the population expansion parameters of the mitochondrial sequences did not show statistically significant support in all lineages (Table 1). This is particularly noteworthy for the B2 lineage, in which the low genetic variability across the Pyrenees point to a strong bottleneck before the colonization of these mountains. However, the extremely low variability of this lineage (only four variable sites; Table 1) surely diminishes the statistical power of the expansion statistics. Additional mitochondrial data or more variable markers should be used to formally test the existence of these demographic expansions.

It is also worth noting that the range expansions hypothesized here could have taken place during particular periods of very favorable conditions, as shown for other species [78]. In the case of the Pyrenean desman, the abundance of humid habitats during the deglaciation periods could have helped to quickly colonize new areas and extend its distribution range from the glacial refugia.

Thus, the evolutionary history of the Pyrenean desman supports the “refugia within refugia” hypothesis [6], which highlights that the Iberian Peninsula and likely other South European peninsulas cannot be regarded as homogeneous refugia but rather as centers of development of multiple refugia that gave rise to distinct evolutionary lineages within many species. Our results further extend this hypothesis by showing that peninsulas would have helped to develop, not only complex isolation mechanisms, but also the whole glacial processes of contraction and dispersal, leaving strong footprints on the genetic structure of endemic species such as the Pyrenean desman. Although these clear genetic traces had been mostly identified in species of continental distribution [7], some of which left distinctive lineages [79] or even species [80,81] in the southern peninsulas, a growing number of endemic or semi-endemic species shows similarly complex population history patterns within the Iberian Peninsula [72,82-85]. We show here that the genetic structure of the Pyrenean desman, a highly specialized mammal, was also affected by the whole glacial processes at a peninsular scale.

### Small influence of the river network on the genetic structure of the Pyrenean desman

Contrary to the expectation that the genetic structure of a species with a semi-aquatic lifestyle and a strong dependence for clean waters, such as the Pyrenean desman, would be highly related to rivers and drainage basins, we found that only a small proportion of its genetic variation can be attributed to the grouping of populations by major river systems. In fact, identical mitochondrial haplotypes can be found at both sides of different mountain ranges, explaining the lack of strong differentiation among basins. Thus, these data allow us to infer that gene flow between basins exists or existed in a not so distant past. In conclusion, the genetic structure of the Pyrenean desman has been more influenced by the history of the Pleistocene glaciations than by its current aquatic habitat distribution, in spite of the strong fragmentation of such specialized habitat. This situation is intermediate between strictly aquatic organisms, whose genetic diversity has been more conditioned by river basins [1], and highly mobile semi-aquatic mammals, such as the Eurasian otter, whose genetic diversity is totally unrelated to river basins [86].

### Strong signatures of isolation in the contact zones

The most unexpected finding in the genetic structure of the Pyrenean desman was the existence of narrow contact zones between the mitochondrial lineages that came into secondary contact after the post-glacial recolonization, with no apparent mixing among them. Actually, dispersal of the four lineages in different directions from the peripheral glacial refugia and the lack of suitable areas in the central parts (Meseta Central) have created an interesting circular distribution of the Pyrenean desman (Figure 9). In addition, the interrupted dispersal of lineage B1 in both clockwise and anti-clockwise directions, and of lineages A1 and A2 towards the areas of lineage B1, have created two prominent genetic gaps, that is, there are two replicate contact zones of the major genetic groups, A and B (Figure 9).

The strongest genetic gap was found in the middle of the Iberian Mountain Range, one of the places where the A and B groups meet (Figure 1A). The 23 samples collected in six rivers of the Iberian Range revealed that individuals belonging to both major lineages were present in this area. However, with the samples available so far, the lineages are segregated and have not been found together in any river stretch. In fact, we can trace a separation line (basically along the valley of the Najerilla river) that seems to restrict the dispersal of female desmans. The second genetic gap was found in the middle of the Cantabrian Mountains and it also affects the same major lineages, A and B. Despite conducting several surveys in this area of the Cantabrian Mountains, we could not get more samples to narrow the closest distance between both lineages. Therefore, we cannot determine at present whether or not some mixing of lineages occurs in this contact zone. However, the lack of penetration of females of one lineage into the distribution area of the other lineage is a remarkable circumstance in both contact zones, where no apparent barriers to dispersal of desmans exist. Although similar situations have been observed in other species [60,66,87-89], including some of the Iberian Peninsula [72,82-84,90,91], certain degree of permeability through the contact zones is normally observed in these species, in contrast with the more strict situation seen in the Pyrenean desman. This phenomenon of competitive exclusion within species could have adaptive or neutral (demographic) causes [92]. Although adaptive processes cannot be excluded, it has been suggested that saturation of the habitat in the contact zones would inhibit female migration in some species (density blocking) [91,93]. This would explain why some of these species have dispersed hundreds of kilometers through empty spaces from glacial refugia but now seem unable to cross a stretch of a few kilometers [92].

The analysis of contact zones discussed so far has been based on mitochondrial data and therefore only refers to the dispersal pattern of females. Although obviously a crucial aspect of the species biology, it may not tell the whole story. In fact, in many species in which nuclear data was obtained, it has been observed that these barriers were not so strong or that they were absent for these markers, indicating male-biased dispersal [83,89,94]. Our intron sequences did not show enough variability within *G. pyrenaicus* to analyze these aspects in depth. However, three variants of the obtained SNPs (Figure 6) exhibited enough geographic extension to be useful in the analysis of dispersal [95]. The three derived mutations showed a contiguous distribution within the sampling localities, suggesting that they arose in place and are of recent origin. In fact, one of the three mutations (in intron *DHRS3-3*; Figure 6) crosses the Cantabrian Mountains contact zone, suggesting that some male-driven dispersal has occurred through it, giving rise to certain degree of introgression. However, additional nuclear genetic data will be necessary to study these aspects in a more quantitative manner. So far, radio-tracking and recapture data of desmans have not revealed sex-biased dispersal [25] but data are still very scarce. Therefore further studies, both genetic and behavioral, should be carried out to better understand the mobility patterns and barriers to dispersal of the Pyrenean desman.

### Subspecies

The existence of two main mitochondrial groups in the Pyrenean desman could in principle correspond to the two described subspecies, *G. p. pyrenaicus* and *G. p. rufulus*, but the distribution of the mitochondrial groups does not perfectly fit with any of the proposed distribution areas for the two subspecies, which have been very unstable in previous works [19-21]. However, none of the previous studies trying to delimit morphologically the subspecies took into account the boundaries between the populations revealed in this work. They rather mixed specimens belonging to different mitochondrial lineages in the analyses. For example, all specimens of the Iberian Range were pooled into a single population when, in fact, there are two distinct lineages in this region. This could have hindered the detection of significant morphological differences between subspecies [19,21]. Future studies aimed at assessing the validity of these subspecies should analyze phenotypic differences between these groups and possible morphological gradients in the contact zones detected in this work. For the moment, according to the genetic results and the corresponding type localities of the subspecies [20], the populations of mitochondrial group A would correspond to subspecies *G. p. rufulus*, and those of group B to subspecies *G. p. pyrenaicus*.

### Implications for conservation of the Pyrenean desman

The Pyrenean desman is legally protected in the four countries where it is present and it was classified as “Vulnerable” in the IUCN Red List [12]. In addition, the populations of the Central Mountain System, in the southern part of the distribution, were recently catalogued by the Spanish Government as “In danger of extinction”, which is the highest protection category. The desman is therefore one of the most threatened mammals of the Iberian Peninsula and, by extension, of the European continent. Indeed, many data seem to indicate a substantial decline of the Pyrenean desman in the Central System in recent times [11]. Actually, our own surveys did not yield any desman excrement in several localities of the Central System where the species had been captured in the last few decades, which forced us to rely on museum samples for our DNA work. More targeted surveys in the most southern parts of the historical range will be of utmost importance in future demographic and genetic studies of the species.

The genetic diversity of the Pyrenean desman was very small in its whole range, as confirmed with both mitochondrial and nuclear markers. Regarding mitochondrial data, for which there are more data for comparison, the nucleotide diversity of the Pyrenean desman is around four times smaller than the mean for mammals [96], and it is particularly low in some areas such as the Pyrenees. Interestingly, however, the Pyrenean populations have been until recently in a relatively good state of conservation [18]. In fact, as we have shown, this low genetic variability was likely due to a recent colonization of the Pyrenees (and not necessarily to a decline of these populations). However, it is important to be aware of the populations with the lowest genetic diversity values in case of future unforeseen environmental changes, which might be more detrimental for them.

The conclusions about the lack of strong genetic differentiation among river basins of the Pyrenean desman may also have implications for conservation purposes. In particular, these results allow us to infer that desmans have not been confined to the river basins where they inhabit and that they can move, or have moved in the recent past, through at least some of the watersheds. Therefore, connectivity between some water basins should not be discarded, in certain cases, to prevent or to reverse an excessive fragmentation of the populations. However, future studies will be necessary to determine the amount of recent gene flow between specific basins in order to properly inform conservation actions in this regard.

A crucial aspect that should be certainly taken into account in conservation programs is the delimitation of *G. pyrenaicus* into the four mitochondrial lineages found in this work. These lineages started to diverge during the Pleistocene glaciations and, in consequence, their integrity should be preserved until further studies establish the exact degree of genetic exchange between these populations [97]. Therefore, following a precautionary principle, these lineages should be considered as different evolutionary units for conservation purposes. In particular, great care should be exercised to avoid any translocation of individuals between these units and thus preserve both the integrity of the Pyrenean desman and its evolutionary history.

## Conclusions

Mitochondrial and nuclear data in the Pyrenean desman (*Galemys pyrenaicus*) allowed us to study the phylogeography of this species and provided evidence for an evolutionary history deeply influenced by the Pleistocene glaciations. One of the most striking findings of this work was the existence of a strong phylogeographic structure in the Pyrenean desman, in which two large groups, A and B, were subdivided into two further groups to give a total of four mitochondrial lineages with parapatric distribution (A1, A2, B1 and B2). Two narrow contact zones between the major groups (A and B), one in the Iberian Range and the other in the Cantabrian Mountains, indicate incomplete mixing after the post-glacial recolonization, at least for females. Nuclear data seem to indicate some degree of gene flow in these contact zones but more data will be necessary to further study the dispersal patterns of the desman. It is interesting to note that the presence of two major and parapatric mitochondrial groups parallels the existence of the two described subspecies, *G. p. pyrenaicus* and *G. p. rufulus*, whose distributions roughly correspond to groups B and A, respectively.

A dating analysis of the desmanines allowed us to estimate that the separation of the major mitochondrial lineages likely occurred in the Middle Pleistocene. In addition, both the geographic variation of genetic diversity (with the populations of highest diversity in the NW part and those of lowest diversity in the Pyrenees) and a species distribution model projected to the LGM coincided in indicating that the most important glacial refugium was in the NW of the Iberian Peninsula. Other minor refugia can be postulated in other parts of the distribution areas of the present mitochondrial lineages. A Holocene expansion from these refugia, but interrupted at the contact zones, led to the current parapatric distribution of the mitochondrial lineages.

The Pyrenean desman is an endangered species and its situation has worsened during the last few years in part of its distribution range, particularly in the most southern populations. In order to undertake the most favorable actions for the long-term survival of this species, conservation programs should keep in mind the peculiar genetic patterns found in this work. Most importantly, artificial mixing of desmans and, particularly, of individuals belonging to different lineages should be avoided. At the moment, almost no natural exchange between the lineages with different glacial origins has been observed and therefore no artificial translocations between them should be carried out until further studies establish the exact degree of genetic exchange between these populations. Although only following these criteria in management plans does not guarantee the conservation of the species, it would be essential to take this information into account in order to prevent an aggravation of the status of this singular species.

### Availability of supporting data

All sequences obtained in this study have been deposited in GenBank under accession numbers JX290581 - JX291096 (see Additional file 1: Table S4). Alignments and trees reconstructed for the different genes of *Galemys*, Laurasiatherians and Talpids have been deposited in TreeBASE under accession number S14084 (http://purl.org/phylo/treebase/phylows/study/TB2:S14084).

## Competing interests

The authors declare that they have no competing interests.

## Authors’ contributions

JI performed sampling and laboratory work, analyzed data, and helped in writing the manuscript. PA, AF-G, JG-E, AG and JG performed sampling, contributed additional samples and information, and helped to interpret the data. JG helped to design the project. RA helped in laboratory work. JC designed the project, performed sampling, analyzed data, and wrote the manuscript. All authors discussed the results and contributed to the preparation of the manuscript. All authors read and approved the final manuscript.

## Supplementary Material

Additional file 1 **Supporting tables and figures. Table S1.** Specimens used in this study. **Table S2.** Primers used for the amplification of three overlapping fragments of the mitochondrial cytochrome *b* gene and a D-loop fragment. **Table S3.** Nuclear markers and primers used in this study. **Table S4.** GenBank accession numbers. **Table S5.** Calibration constraints (in Myr) used as priors in the BEAST analysis of Laurasiatherian mammals. **Figure S1.** Contour plot of genetic diversity (π) of *Galemys pyrenaicus* in which samples of populations in secondary contact were mixed in the calculations. Click here for file
