# Supplementary material for: Phylogeography and postglacial expansion of the endangered semi-aquatic mammal Galemys pyrenaicus
Source: BMC Evol Biol. 2013 Jun 6;13:115. doi: 10.1186/1471-2148-13-115 (PMC3682870; doi:10.1186/1471-2148-13-115)
Supplement: Additional file 1 — Supporting tables and figures. Table S1. Specimens used in this study. Table S2. Primers used for the amplification of three overlapping fragments of the mitochondrial cytochrome b gene and a D-loop fragment. Table S3. Nuclear markers and primers used in this study. Table S4. GenBank accession numbers. Table S5. Calibration constraints (in Myr) used as priors in the BEAST analysis of Laurasiatherian mammals. Figure S1. Contour plot of genetic diversity (π) of Galemys pyrenaicus in which samples of populations in secondary contact were mixed in the calculations. [file 1471-2148-13-115-S1.pdf]

Table S1. Specimens used in this study

Specimen Code	Sample Type	Locality	Basin	Latitude	Longitude
IBE-C216	Faeces	Ars	Ebro	42.4	1.4
IBE-C224	Faeces	Ars	Ebro	42.4	1.4
IBE-C320	Tissue	Tor	Ebro	42.6	1.4
IBE-C321	Tissue	Tor	Ebro	42.6	1.4
IBE-C421	Tissue	Tor	Ebro	42.6	1.4
IBE-C422	Tissue	Tor	Ebro	42.6	1.4
IBE-C428	Tissue	Tor	Ebro	42.6	1.4
IBE-C437	Tissue	Barriomartin	Duero	42.0	-2.5
IBE-C438	Tissue	San Emiliano	Duero	43.0	-6.0
IBE-C448	Tissue	Rio Salentinos-Sil	Miño	42.8	-6.4
IBE-C449	Tissue	Rio Somiedo-Somiedo	Cantabrian Basins	43.1	-6.3
IBE-C452	Tissue	Rio Ambroz-Hervas	Tajo	40.3	-5.9
IBE-C485	Tissue	Rio Ambroz-Hervas	Tajo	40.3	-5.8
IBE-C539	Faeces	Confluencia Deva-Cicera	Cantabrian Basins	43.2	-4.6
IBE-C600	Faeces	Toran	Garona	42.8	0.8
IBE-C618	Faeces	Rio Besaya-Ventorrillo	Cantabrian Basins	43.1	-4.1
IBE-C660	Tissue	Ouro Zona Baja-Tamega	Duero	41.5	-7.8
IBE-C683	Faeces	Rio Besaya-Ventorrillo	Cantabrian Basins	43.1	-4.1
IBE-C720	Faeces	Cantijan	Cantabrian Basins	43.1	-4.8
IBE-C723	Faeces	Deva	Cantabrian Basins	43.1	-4.8
IBE-C737	Faeces	Deva	Cantabrian Basins	43.1	-4.8
IBE-C779	Faeces	Cabecera Douro-Tamega	Duero	41.6	-8.0
IBE-C802	Faeces	Lousas-Zona Baja Beca-Tamega	Duero	41.6	-7.8
IBE-C831	Faeces	Gondiaes-Beca-Tamega	Duero	41.6	-7.9
IBE-C844	Faeces	Oura	Duero	41.7	-7.5
IBE-C861	Faeces	Oura	Duero	41.7	-7.5
IBE-C894	Faeces	Ouro-Tamega	Duero	41.5	-7.8
IBE-C895	Faeces	Avelares-Louredo-Tamega	Duero	41.5	-7.8
IBE-C919	Tissue	Ouro Zona Baja	Duero	41.5	-7.8
IBE-C922	Faeces	Louredo-Tamega	Duero	41.5	-7.9
IBE-C964	Faeces	Toran	Garona	42.8	0.8
IBE-C975	Faeces	Rio Puerma-Yuso	Duero	43.1	-4.9
IBE-C979	Faeces	Rio Puerma-Yuso	Duero	43.1	-4.9
IBE-C982	Faeces	Escrita	Ebro	42.6	1.0
IBE-C990	Faeces	Varrados	Garona	42.8	0.8
IBE-C1002	Faeces	Puerto de Canto	Ebro	42.4	1.2
IBE-C1053	Faeces	Ronas-Najerilla-Anguiano	Ebro	42.2	-2.8
IBE-C1068	Faeces	Portilla-Najerilla-Villavelayo	Ebro	42.1	-2.9
IBE-C1069	Tissue	Rio Calamantio-Mansilla de la Sierra	Ebro	42.2	-2.9
IBE-C1070	Faeces	Arroyo Usaya y Oja-Ezcaray	Ebro	42.3	-3.0
IBE-C1072	Faeces	Arroyo Ortigal-Oja-Ezcaray	Ebro	42.2	-3.1
IBE-C1075	Faeces	Oja y Arroyo Altuzarra-Ezcaray	Ebro	42.2	-3.0
IBE-C1077	Faeces	Arroyo Usaya y Oja-Ezcaray	Ebro	42.3	-3.0
IBE-C1132	Faeces	Rio Razoncillo-Molinos de Razon	Duero	42.0	-2.6
IBE-C1154	Faeces	Rio del Oro-Tremado	Cantabrian Basins	43.2	-6.8
IBE-C1174	Faeces	Rio Valledor-Villanueva	Cantabrian Basins	43.1	-6.8
IBE-C1177	Faeces	Rio Valledor-Villanueva	Cantabrian Basins	43.1	-6.8
IBE-C1611	Faeces	Rio Ter-Pastiura	Catalan Basins	42.4	2.3
IBE-C1612	Faeces	Riu Ritort-Espinavell	Catalan Basins	42.4	2.4
IBE-C1661	Faeces	Rio Razon-Sotillo del Rincon	Duero	41.9	-2.7
IBE-C1671	Faeces	Rio Razon-Sotillo del Rincon	Duero	41.9	-2.7
IBE-C1687	Faeces	Rio Razoncillo-Molinos de Razon	Duero	42.0	-2.6
IBE-C1729	Faeces	Entrambasaguas-Rio Hijar	Ebro	43.0	-4.3
IBE-C1843	Faeces	Rio del Coto-Arroyo de la Brana	Cantabrian Basins	43.1	-6.7
IBE-C1851	Faeces	Rio Deva-Convento del Naranco	Cantabrian Basins	43.1	-4.8
IBE-C1869	Faeces	Rio de Pumarin-Pontenova	Cantabrian Basins	43.3	-6.7
IBE-C1880	Faeces	Rio Cantijan-Puente Pontesque	Cantabrian Basins	43.1	-4.8
IBE-C2517	Faeces	Redes-Caleao	Cantabrian Basins	43.2	-5.4
IBE-C2596	Faeces	Rio Valvanera-Monasterio	Ebro	42.2	-2.9
IBE-C2597	Faeces	Rio Cidacos-Peroblasco	Ebro	42.2	-2.3
IBE-C2607	Faeces	Redes-Pendones	Cantabrian Basins	43.1	-5.3
IBE-C2736	Tissue	Rio Caranyo-Covelo	Miño	42.3	-8.4
IBE-C2737	Tissue	Rio Riobo-A Estrada	Galician Basins	42.7	-8.4
IBE-C2738	Tissue	Rio Riobo-A Estrada	Galician Basins	42.7	-8.4
IBE-C2739	Tissue	Rio Cabras-Laza	Duero	42.1	-7.4
IBE-C2740	Tissue	Rio Meladas-Carballeda	Miño	42.2	-6.8
IBE-C2741	Tissue	Rio Meladas-Carballeda	Miño	42.2	-6.8
IBE-C2742	Tissue	Rio Ourille-Celanova	Miño	42.2	-7.9
IBE-C2743	Tissue	Rio Ourille-Celanova	Miño	42.2	-7.9
IBE-C2744	Tissue	Rio Pontigon-Fonsagrada	Cantabrian Basins	43.2	-7.0
IBE-C2745	Tissue	Rio Pontigon-Fonsagrada	Cantabrian Basins	43.2	-7.0
IBE-C2746	Tissue	Rio Queixa-Chandrexa de Queixa	Miño	42.2	-7.4
IBE-C2747	Tissue	Rio Queixa-Chandrexa de Queixa	Miño	42.2	-7.4
IBE-C2749	Tissue	Rio Termes-As Neves	Miño	42.1	-8.4
IBE-C2750	Tissue	Rio Termes-As Neves	Miño	42.1	-8.4
IBE-C2751	Tissue	Rio Ribeira Pequena-Laza	Miño	42.1	-7.4
IBE-C2752	Tissue	Rio Ribeira Pequena-Laza	Miño	42.1	-7.4
IBE-C2753	Tissue	Rio Bullan-Navia de Suarna	Cantabrian Basins	42.9	-7.1
IBE-C2754	Tissue	Rio Marinan-Obarco	Miño	42.4	-7.0
IBE-C2755	Tissue	Rio Leitzaran-Berastegi	Cantabrian Basins	43.2	-2.0
IBE-C2756	Tissue	Rio Aiaiturrieta-Ataun	Cantabrian Basins	43.0	-2.1
IBE-C2757	Tissue	Rio Amundarain-Zaldibia	Cantabrian Basins	43.1	-2.0
IBE-C2760	Tissue	Rio Erasote-Leitza	Cantabrian Basins	43.1	-1.9
IBE-C2763	Tissue	Rio Olazar-Eugi	Ebro	43.0	-1.5
IBE-C2765	Tissue	Rio Ezpelura-Urrotz	Cantabrian Basins	43.1	-1.7
IBE-C2781	Tissue	Rio Ameztia-Labaien	Cantabrian Basins	43.1	-1.7

IBE-C2791	Tissue	Rio Urrobi-Auritz	Ebro	43.0	-1.3
IBE-C2795	Tissue	Rio Irati-Artozki	Ebro	42.9	-1.3
IBE-C2796	Tissue	Rio Sasoaran-Eugi	Ebro	43.0	-1.6
IBE-C2798	Tissue	Rio Arrazola-Orbaitzeta	Ebro	43.0	-1.2
IBE-C2799	Tissue	Rio Elama-Artikutza	Cantabrian Basins	43.2	-1.8
IBE-C2856	Tissue	Rio Lamedo	Cantabrian Basins	43.1	-4.6
IBE-C2892	Tissue	Rio Cares-Cain	Cantabrian Basins	43.2	-4.9
IBE-C2893	Tissue	Rio Cares-Corona	Cantabrian Basins	43.2	-4.9
IBE-C2894	Tissue	Rio Cares-Casiellos	Cantabrian Basins	43.2	-4.9
IBE-C2950	Faeces	Redes-Caleao	Cantabrian Basins	43.2	-5.4
IBE-C2975	Faeces	Ribeira do Castelo	Duero	41.6	-7.7
IBE-C2990	Faeces	Lousas-Cabecera Beca-Tamega	Duero	41.6	-7.8
IBE-C3159	Claw (EBD ADN1254)	Rio Lillas-Cantalojas	Tajo	41.2	-3.2
IBE-C3161	Rib fragment (EBD ADN1256)	Navacepeda de Tormes	Duero	40.4	-5.2
IBE-C3301	Tissue	Paiva-Fraguas	Duero	40.8	-7.8
IBE-C3303	Tissue	Sabor-Macas-Quintanilla	Duero	41.8	-6.6
IBE-C3305	Tissue	Paiva-Fraguas	Duero	40.8	-7.8
IBE-C3338	Faeces	Rio Bullon	Cantabrian Basins	43.1	-4.5
IBE-C3342	Faeces	Rio Bullon-Valdeprado	Cantabrian Basins	43.1	-4.5
IBE-C3455	Faeces	Pedroso-Najerilla-Pedroso	Ebro	42.3	-2.7
IBE-C3473	Faeces	Arroyo de Puente la Ra-Iregua	Ebro	42.1	-2.7
IBE-C3474	Faeces	Tobia-Najerilla	Ebro	42.2	-2.9
IBE-C3475	Faeces	Tobia-Najerilla	Ebro	42.3	-2.9
IBE-C3481	Faeces	Tobia-Najerilla	Ebro	42.3	-2.9
IBE-C3482	Faeces	Lumbreras-Iregua-Lumbreras	Ebro	42.1	-2.6
IBE-C3486	Faeces	Arroyo la Vieja-Iregua-Lumbreras	Ebro	42.0	-2.6
IBE-C3489	Faeces	Arroyo la Vieja-Iregua-Lumbreras	Ebro	42.0	-2.6
IBE-C3522	Tissue	Sabor-Franca-Soutelo	Duero	41.9	-6.8
IBE-C3541	Tissue	Sabor-Abaixo de Gimonde	Duero	41.8	-6.7
IBE-C3545	Tissue	Sabor-Franca-Soutelo	Duero	41.9	-6.8
IBE-C3602	Tissue	Corgo-Cabril-Arnal	Duero	41.3	-7.8
IBE-C3620	Faeces	Curueno	Duero	42.9	-5.4
IBE-C3623	Faeces	Redes-Caleao	Cantabrian Basins	43.2	-5.4
IBE-C3637	Tissue	Sabor-Franca-Soutelo	Duero	41.9	-6.8
IBE-C3638	Tissue	Sabor-Franca-Soutelo	Duero	41.9	-6.8
IBE-C3641	Faeces	Redes-Monasterio	Cantabrian Basins	43.2	-5.3
IBE-C3647	Tissue	Sabor-Franca-Soutelo	Duero	41.9	-6.8
IBE-C3756	Tissue	La Soledad	Duero	42.2	-3.1
IBE-C3759	Tissue	Urumea	Cantabrian Basins	43.1	-1.8
IBE-C3762	Tissue	Luzaide	Adour	43.0	-1.3
IBE-C3763	Tissue	Aritzakun	Adour	43.3	-1.4
IBE-S1925	Tissue	Riu Ritort-Camprodon	Catalan Basins	42.3	2.4
IBE-S2012	Faeces	Riu del Canto-Soriguera	Ebro	42.4	1.2
IBE-S2026	Faeces	Riu del Canto-Soriguera	Ebro	42.4	1.2
IBE-S2762	Faeces	Hecho-Aragon-Subordan	Ebro	42.8	-0.6
IBE-S2764	Faeces	Hecho-Aragon-Subordan	Ebro	42.9	-0.7
IBE-S2768	Faeces	Hecho-Aragon-Subordan	Ebro	42.9	-0.7
IBE-S2773	Faeces	Hecho-Aragon-Subordan	Ebro	42.8	-0.7

Table S2. Primers used for the amplification of three overlapping fragments of the mitochondrial cytochrome *b* gene and a D-loop fragment.

Marker	Primer sequences	Length
cytochrome *b*	ACTAATGACATGAAAAATCATCGTT / TACAAGATCAGTTCCGATGTAAG	483
cytochrome *b*	TCTTACCATGGGGTCAAATATC / AGATAATTAGAATGAGGATTAGTG	278
cytochrome *b*	GGATTATCATCCGACACTGATAA / TTTTCGTTTTTGGTTTACAAGAC	516
D-loop	CCGCCACCAACACCCAAAGCTG / GATGGTAGTCGAGAGATAACCT	367

Table S3. Nuclear intron markers and primers used in this study.

Marker	Primer sequences	T (ºC)	Length	Differences *Galemys* - *Desmana*
ACOX2-3	CCTSGGCTCDGAGGAGCAGAT / GGGCTGTGHAYCACAAACTCCT	60	381	15
ACPT-4	GAYTTTGACCGSACVCTGGAGAG / AGYAGYTCVYGGTATCGRGGACA	60	279	28
COPS7A-4	TACAGCATYGGRCGRGACATCCA / TCACYTGCTCCTCRATGCCKGACA	62	634	23
DHRS3-3	GACATCACCATCCTGGTGAACAA / AGGGTCAGGCTCTCCATGAAG	TD	259	27
LANCL1-4	TTTGGARWRGARAARATTCCTCA / GRTACCAYTCRTACAWCAGTGG	TD	523	25
PRPF31-3	GTCATYGTRGAYGCYAACAAC / BTTSACNGTGCGGATGTAATC	61	487	28
ROGDI-7	CTGATGGAYGCYGTGATGCTGCA / CACGGTGAGGCASAGCTTGTTGA	60	291	20
SMYD4-5	CAGCCTYCTGAAYCAYTCCTG / CAGBNRCAGTCAAAGAARTACTG	TD	402	15

TD: touchdown PCR in which the annealing temperature was lowered from 65 ºC to 50 ºC at 1 ºC decrease per cycle.

Table S4. GenBank accession numbers

Specimen Code	Species	cytochrome *b*	D-loop (1-342)	ACOX2-3	ACPT-4	COPS7A-4	DHRS3-3	LANCL1-4	PRPF31-3	ROGDI-7	SMYD4-5
IBE-C216	*Galemys pyrenaicus*	JX290604	JX290738								
IBE-C224	*Galemys pyrenaicus*	JX290605	JX290739								
IBE-C320	*Galemys pyrenaicus*	JX290649	JX290783								
IBE-C321	*Galemys pyrenaicus*	JX290650	JX290784								
IBE-C421	*Galemys pyrenaicus*	JX290678	JX290812	JX290963	JX291006	JX291056	JX290933	JX290870	JX291086	JX290903	JX290994
IBE-C422	*Galemys pyrenaicus*	JX290679	JX290813								
IBE-C428	*Galemys pyrenaicus*	JX290680	JX290814	JX290964	JX291007	JX291057	JX290934	JX290871	JX291087	JX290904	JX290995
IBE-C437	*Galemys pyrenaicus*	JX290681	JX290815								
IBE-C438	*Galemys pyrenaicus*	JX290682	JX290816								
IBE-C448	*Galemys pyrenaicus*	JX290683	JX290817	JX290965	JX291008	JX291058	JX290935	JX290872	JX291088	JX290905	JX290996
IBE-C449	*Galemys pyrenaicus*	JX290684	JX290818	JX290966	JX291009	JX291059	JX290936	JX290873	JX291089	JX290906	JX290997
IBE-C452	*Galemys pyrenaicus*	JX290685	JX290819	JX290967	JX291010	JX291060	JX290937	JX290874	JX291090	JX290907	JX290998
IBE-C485	*Galemys pyrenaicus*	JX290686	JX290820	JX290968	JX291034	JX291061	JX290938	JX290875	JX291091	JX290908	JX290999
IBE-C539	*Galemys pyrenaicus*	JX290687	JX290821								
IBE-C600	*Galemys pyrenaicus*	JX290688	JX290822								
IBE-C618	*Galemys pyrenaicus*	JX290689	JX290823								
IBE-C660	*Galemys pyrenaicus*	JX290690	JX290824	JX290969	JX291029	JX291062	JX290939	JX290876	JX291092	JX290909	JX291000
IBE-C683	*Galemys pyrenaicus*	JX290691	JX290825								
IBE-C720	*Galemys pyrenaicus*	JX290692	JX290826								
IBE-C723	*Galemys pyrenaicus*	JX290693	JX290827								
IBE-C737	*Galemys pyrenaicus*	JX290694	JX290828								
IBE-C779	*Galemys pyrenaicus*	JX290695	JX290829								
IBE-C802	*Galemys pyrenaicus*	JX290696	JX290830								
IBE-C831	*Galemys pyrenaicus*	JX290697	JX290831								
IBE-C844	*Galemys pyrenaicus*	JX290698	JX290832								
IBE-C861	*Galemys pyrenaicus*	JX290699	JX290833								
IBE-C894	*Galemys pyrenaicus*	JX290700	JX290834								
IBE-C895	*Galemys pyrenaicus*	JX290701	JX290835								
IBE-C919	*Galemys pyrenaicus*	JX290702	JX290836	JX290970	JX291030	JX291063	JX290940	JX290877	JX291093	JX290910	JX291001
IBE-C922	*Galemys pyrenaicus*	JX290703	JX290837								
IBE-C964	*Galemys pyrenaicus*	JX290704	JX290838								
IBE-C975	*Galemys pyrenaicus*	JX290705	JX290839								
IBE-C979	*Galemys pyrenaicus*	JX290706	JX290840								
IBE-C982	*Galemys pyrenaicus*	JX290707	JX290841								
IBE-C990	*Galemys pyrenaicus*	JX290708	JX290842								
IBE-C1002	*Galemys pyrenaicus*	JX290582	JX290716								
IBE-C1053	*Galemys pyrenaicus*	JX290583	JX290717								
IBE-C1068	*Galemys pyrenaicus*	JX290584	JX290718								
IBE-C1069	*Galemys pyrenaicus*	JX290585	JX290719	JX290943	JX291027	JX291036	JX290913	JX290850	JX291066	JX290883	JX290974

IBE-C1070	*Galemys pyrenaicus*	JX290586	JX290720								
IBE-C1072	*Galemys pyrenaicus*	JX290587	JX290721								
IBE-C1075	*Galemys pyrenaicus*	JX290588	JX290722								
IBE-C1077	*Galemys pyrenaicus*	JX290589	JX290723								
IBE-C1132	*Galemys pyrenaicus*	JX290590	JX290724								
IBE-C1154	*Galemys pyrenaicus*	JX290591	JX290725								
IBE-C1174	*Galemys pyrenaicus*	JX290592	JX290726								
IBE-C1177	*Galemys pyrenaicus*	JX290593	JX290727								
IBE-C1611	*Galemys pyrenaicus*	JX290594	JX290728								
IBE-C1612	*Galemys pyrenaicus*	JX290595	JX290729								
IBE-C1661	*Galemys pyrenaicus*	JX290596	JX290730								
IBE-C1671	*Galemys pyrenaicus*	JX290597	JX290731								
IBE-C1687	*Galemys pyrenaicus*	JX290598	JX290732								
IBE-C1729	*Galemys pyrenaicus*	JX290599	JX290733								
IBE-C1843	*Galemys pyrenaicus*	JX290600	JX290734								
IBE-C1851	*Galemys pyrenaicus*	JX290601	JX290735								
IBE-C1869	*Galemys pyrenaicus*	JX290602	JX290736								
IBE-C1880	*Galemys pyrenaicus*	JX290603	JX290737								
IBE-C2517	*Galemys pyrenaicus*	JX290606	JX290740								
IBE-C2596	*Galemys pyrenaicus*	JX290607	JX290741								
IBE-C2597	*Galemys pyrenaicus*	JX290608	JX290742								
IBE-C2607	*Galemys pyrenaicus*	JX290609	JX290743								
IBE-C2736	*Galemys pyrenaicus*	JX290610	JX290744								
IBE-C2737	*Galemys pyrenaicus*	JX290611	JX290745	JX290944	JX291012	JX291037	JX290914	JX290851	JX291067	JX290884	JX290975
IBE-C2738	*Galemys pyrenaicus*	JX290612	JX290746								
IBE-C2739	*Galemys pyrenaicus*	JX290613	JX290747	JX290945	JX291019	JX291038	JX290915	JX290852	JX291068	JX290885	JX290976
IBE-C2740	*Galemys pyrenaicus*	JX290614	JX290748	JX290946	JX291013	JX291039	JX290916	JX290853	JX291069	JX290886	JX290977
IBE-C2741	*Galemys pyrenaicus*	JX290615	JX290749								
IBE-C2742	*Galemys pyrenaicus*	JX290616	JX290750								
IBE-C2743	*Galemys pyrenaicus*	JX290617	JX290751	JX290947	JX291025	JX291040	JX290917	JX290854	JX291070	JX290887	JX290978
IBE-C2744	*Galemys pyrenaicus*	JX290618	JX290752								
IBE-C2745	*Galemys pyrenaicus*	JX290619	JX290753								
IBE-C2746	*Galemys pyrenaicus*	JX290620	JX290754								
IBE-C2747	*Galemys pyrenaicus*	JX290621	JX290755								
IBE-C2749	*Galemys pyrenaicus*	JX290622	JX290756	JX290948	JX291014	JX291041	JX290918	JX290855	JX291071	JX290888	JX290979
IBE-C2750	*Galemys pyrenaicus*	JX290623	JX290757								
IBE-C2751	*Galemys pyrenaicus*	JX290624	JX290758	JX290949	JX291015	JX291042	JX290919	JX290856	JX291072	JX290889	JX290980
IBE-C2752	*Galemys pyrenaicus*	JX290625	JX290759								
IBE-C2753	*Galemys pyrenaicus*	JX290626	JX290760	JX290950	JX291020	JX291043	JX290920	JX290857	JX291073	JX290890	JX290981
IBE-C2754	*Galemys pyrenaicus*	JX290627	JX290761	JX290951	JX291026	JX291044	JX290921	JX290858	JX291074	JX290891	JX290982
IBE-C2755	*Galemys pyrenaicus*	JX290628	JX290762	JX290952	JX291021	JX291045	JX290922	JX290859	JX291075	JX290892	JX290983
IBE-C2756	*Galemys pyrenaicus*	JX290629	JX290763	JX290953	JX291022	JX291046	JX290923	JX290860	JX291076	JX290893	JX290984

IBE-C2757	*Galemys pyrenaicus*	JX290630	JX290764	JX290954	JX291023	JX291047	JX290924	JX290861	JX291077	JX290894	JX290985
IBE-C2760	*Galemys pyrenaicus*	JX290631	JX290765								
IBE-C2763	*Galemys pyrenaicus*	JX290632	JX290766	JX290955	JX291016	JX291048	JX290925	JX290862	JX291078	JX290895	JX290986
IBE-C2765	*Galemys pyrenaicus*	JX290633	JX290767	JX290956	JX291017	JX291049	JX290926	JX290863	JX291079	JX290896	JX290987
IBE-C2781	*Galemys pyrenaicus*	JX290634	JX290768								
IBE-C2791	*Galemys pyrenaicus*	JX290635	JX290769								
IBE-C2795	*Galemys pyrenaicus*	JX290636	JX290770	JX290957	JX291018	JX291050	JX290927	JX290864	JX291080	JX290897	JX290988
IBE-C2796	*Galemys pyrenaicus*	JX290637	JX290771								
IBE-C2798	*Galemys pyrenaicus*	JX290638	JX290772								
IBE-C2799	*Galemys pyrenaicus*	JX290639	JX290773	JX290958	JX291024	JX291051	JX290928	JX290865	JX291081	JX290898	JX290989
IBE-C2856	*Galemys pyrenaicus*	JX290640	JX290774	JX290959	JX291028	JX291052	JX290929	JX290866	JX291082	JX290899	JX290990
IBE-C2892	*Galemys pyrenaicus*	JX290641	JX290775	JX290960	JX291033	JX291053	JX290930	JX290867	JX291083	JX290900	JX290991
IBE-C2893	*Galemys pyrenaicus*	JX290642	JX290776	JX290961	JX291031	JX291054	JX290931	JX290868	JX291084	JX290901	JX290992
IBE-C2894	*Galemys pyrenaicus*	JX290643	JX290777	JX290962	JX291032	JX291055	JX290932	JX290869	JX291085	JX290902	JX290993
IBE-C2950	*Galemys pyrenaicus*	JX290644	JX290778								
IBE-C2975	*Galemys pyrenaicus*	JX290645	JX290779								
IBE-C2990	*Galemys pyrenaicus*	JX290646	JX290780								
IBE-C3159	*Galemys pyrenaicus*	JX290647	JX290781								
IBE-C3161	*Galemys pyrenaicus*	JX290648	JX290782								
IBE-C3301	*Galemys pyrenaicus*	JX290651	JX290785								
IBE-C3303	*Galemys pyrenaicus*	JX290652	JX290786								
IBE-C3305	*Galemys pyrenaicus*	JX290653	JX290787								
IBE-C3338	*Galemys pyrenaicus*	JX290654	JX290788								
IBE-C3342	*Galemys pyrenaicus*	JX290655	JX290789								
IBE-C3455	*Galemys pyrenaicus*	JX290656	JX290790								
IBE-C3473	*Galemys pyrenaicus*	JX290657	JX290791								
IBE-C3474	*Galemys pyrenaicus*	JX290658	JX290792								
IBE-C3475	*Galemys pyrenaicus*	JX290659	JX290793								
IBE-C3481	*Galemys pyrenaicus*	JX290660	JX290794								
IBE-C3482	*Galemys pyrenaicus*	JX290661	JX290795								
IBE-C3486	*Galemys pyrenaicus*	JX290662	JX290796								
IBE-C3489	*Galemys pyrenaicus*	JX290663	JX290797								
IBE-C3522	*Galemys pyrenaicus*	JX290664	JX290798								
IBE-C3541	*Galemys pyrenaicus*	JX290665	JX290799								
IBE-C3545	*Galemys pyrenaicus*	JX290666	JX290800								
IBE-C3602	*Galemys pyrenaicus*	JX290667	JX290801								
IBE-C3620	*Galemys pyrenaicus*	JX290668	JX290802								
IBE-C3623	*Galemys pyrenaicus*	JX290669	JX290803								
IBE-C3637	*Galemys pyrenaicus*	JX290670	JX290804								
IBE-C3638	*Galemys pyrenaicus*	JX290671	JX290805								
IBE-C3641	*Galemys pyrenaicus*	JX290672	JX290806								
IBE-C3647	*Galemys pyrenaicus*	JX290673	JX290807								

IBE-C3756	*Galemys pyrenaicus*	JX290674	JX290808								
IBE-C3759	*Galemys pyrenaicus*	JX290675	JX290809								
IBE-C3762	*Galemys pyrenaicus*	JX290676	JX290810								
IBE-C3763	*Galemys pyrenaicus*	JX290677	JX290811								
IBE-S1925	*Galemys pyrenaicus*	JX290709	JX290843	JX290971	JX291011	JX291064	JX290941	JX290878	JX291094	JX290911	JX291002
IBE-S2012	*Galemys pyrenaicus*	JX290710	JX290844								
IBE-S2026	*Galemys pyrenaicus*	JX290711	JX290845								
IBE-S2762	*Galemys pyrenaicus*	JX290712	JX290846								
IBE-S2764	*Galemys pyrenaicus*	JX290713	JX290847								
IBE-S2768	*Galemys pyrenaicus*	JX290714	JX290848								
IBE-S2773	*Galemys pyrenaicus*	JX290715	JX290849								
IBE-C3164	*Desmana moschata*	JX290581		JX290972	JX291004	JX291065	JX290912	JX290879	JX291095	JX290882	JX291003
IBE-C686	*Talpa occidentalis*			JX290973	JX291005	JX291035	JX290942	JX290880	JX291096	JX290881	

Table S5. Calibration constraints (in Myr) used as priors in the BEAST analysis of Laurasiatherian mammals.

Clade (Node number)	Minimum hard bound	Maximum soft bound	Lognormal parameters	
			Mean	Offset
Laurasiatheria (1)	62.50	131.50	21.95	62.50
Ferungulata (2)	62.50	131.50	21.95	62.50
Cetartiodactyla (3)	52.40	65.80	4.27	52.40
Cetruminantia (4)	52.40	65.80	4.27	52.40
Zooamata (5)	62.50	131.50	21.95	62.50
Carnivora (6)	39.68	65.80	8.28	39.68

Figure S1

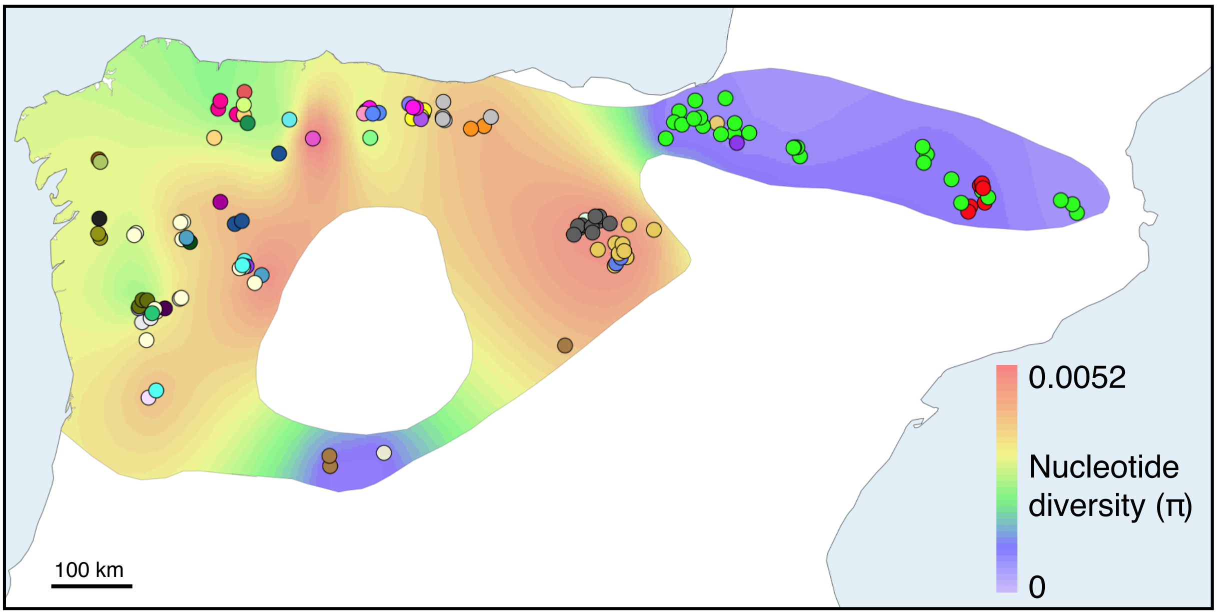

Figure S1 - Contour plot of genetic diversity (π) of *Galemys pyrenaicus* in which samples of populations in secondary contact were mixed in the calculations. Colors of the contour plot indicate interpolated genetic diversity. The contour plot is only drawn for the species distribution area. Sample points have been randomly repositioned within a circle of 5 km radius to reveal samples of the same location. A different color is used for each haplotype and therefore a greater variety of colors in an area also indicates higher genetic diversity.
